# Greetings from the head office!

**DOI:** 10.4103/0972-124X.60222

**Published:** 2009

**Authors:** M. M. Dayakar

**Affiliations:** *Department of Periodontics, K.V.G. Dental College, Sullia, Mangalore - 574 327, Karnataka, India. E-mail: mmdayakar@yahoo.com*

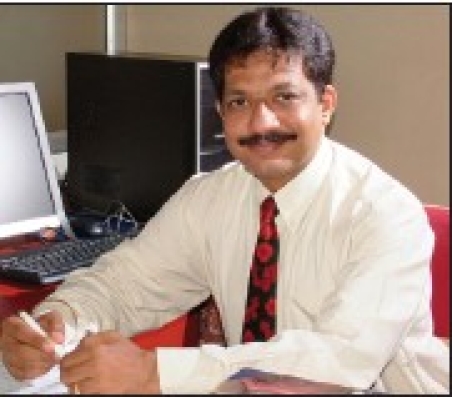



It is my pleasure to share a few thoughts regarding our JISP. A journal is the mouthpiece of any organization. Our journal has gained momentum after the change of guard at the helm as Dr Arunachalam took over as the editor. He is putting great efforts in publishing the journals at regular intervals in spite of shortage of sponsorship. Our society, with various academic as well as association activities, has earned a name of its own and has a positive impact on the dental fraternity. As the president of our society, eminent academician Dr. Dwarakanath has made his intensions clear that he will be focusing on various scientific projects. Our journal will be a platform for the periodontists of our country to publish their research.

I wish you all a very happy New Year ahead. We, at ISP, believe in the principle of moving forward in spite of transient ups and downs.

I thank all the members of the editorial board for their efforts and wish them all the best.

